# Validity and Reproducibility of a Self-Administered Semi-Quantitative Food-Frequency Questionnaire for Estimating Usual Daily Fat, Fibre, Alcohol, Caffeine and Theobromine Intakes among Belgian Post-Menopausal Women

**DOI:** 10.3390/ijerph6010121

**Published:** 2009-01-06

**Authors:** Selin Bolca, Inge Huybrechts, Mia Verschraegen, Stefaan De Henauw, Tom Van de Wiele

**Affiliations:** 1 Laboratory of Microbial Ecology and Technology (LabMET), Faculty of Bioscience Engineering, Ghent University, Coupure Links 653, B-9000 Ghent, Belgium; 2 Department of Public Health, Ghent University, University Hospital 2BlokA, De Pintelaan 185, B-9000 Ghent, Belgium; 3 Department of Nutrition and Dietetics, Faculty of Health Care Vesalius, University College Ghent, Keramiekstraat 80, B-9000 Ghent, Belgium

**Keywords:** Food-frequency questionnaire, estimated diet record, validity, reproducibility, fat, fibre

## Abstract

A novel food-frequency questionnaire (FFQ) was developed and validated to assess the usual daily fat, saturated, mono-unsaturated and poly-unsaturated fatty acid, fibre, alcohol, caffeine, and theobromine intakes among Belgian post-menopausal women participating in dietary intervention trials with phyto-oestrogens. The relative validity of the FFQ was estimated by comparison with 7 day (d) estimated diet records (EDR, *n* 64) and its reproducibility was evaluated by repeated administrations 6 weeks apart (*n* 79). Although the questionnaire underestimated significantly all intakes compared to the 7 d EDR, it had a good ranking ability (*r* 0.47–0.94; weighted κ 0.25–0.66) and it could reliably distinguish extreme intakes for all the estimated nutrients, except for saturated fatty acids. Furthermore, the correlation between repeated administrations was high (*r* 0.71–0.87) with a maximal misclassification of 7% (weighted κ 0.33–0.80). In conclusion, these results compare favourably with those reported by others and indicate that the FFQ is a satisfactorily reliable and valid instrument for ranking individuals within this study population.

## 1. Introduction

The consumption of fat, fibres, alcoholic and caffeinated beverages has been associated with the bioactivation of phyto-oestrogens [1–4], whereas the intake of theobromine (3, 7-dimethylxanthine), a constituent of cacao structurally similar to caffeine (1, 3, 7-trimethylxanthine), might also be important. Phyto-oestrogens are polyphenolic non-steroidal plant derived metabolites, present in the Western diet predominantly as isoflavones, lignans, and prenylflavonoids [5]. In addition to their oestrogen agonistic and antagonistic properties, these compounds exert other non-hormonal effects *in vitro*, such as anti-oxidant [6], chemopreventive [7, 8] and anti-androgenic [9, 10] activities. In the colon several microbial transformations can occur resulting in more biologically active metabolites [11], such as equol [12] and 8-prenylnaringenin [13].

In the context of dietary intervention trials investigating the relation between the background diet and the microbial metabolism of phyto-oestrogens in post-menopausal women [14, 15], an instrument estimating the usual daily total fat, saturated, mono-, and poly-unsaturated fatty acid (SFA, MUFA, and PUFA), fibre, alcohol, caffeine, and theobromine intakes of the participants as accurately and precisely as possible, taking into account feasibility aspects such as respondent burden, was needed. At study onset, no such tool was available. Biochemical parameters reflecting dietary intakes are valuable since they do not rely on self-reports of consumption, but no reliable biomarkers representing long-term intake of total fat [16], fibres, alcohol, caffeine, and theobromine were described, and therefore we had to look for a dietary assessment method. A food-frequency questionnaire (FFQ) was preferred over diet records or 24-h recalls because of its relatively low respondent burden and costs. Jain *et al.* [17] validated a FFQ estimating fat (total fat, SFA, MUFA, and PUFA), fibre, alcohol, and caffeine intakes among Canadian women (54±14 years of age), but theobromine was not considered. Moreover, the performance of a dietary assessment instrument depends on the characteristics and unique dietary features of a population group, limiting its applicability in another group.

A novel self-administered semi-quantitative FFQ was designed to measure the usual daily intakes of total fat, SFA, MUFA, PUFA, fibres, alcohol, caffeine, and theobromine among Belgian women aged between 45 and 75 years old. In order to ensure proper interpretation of the results obtained with this new instrument, a study was conducted 1) to investigate the relative validity using 7 d estimated diet records (EDR) as standard method and 2) to evaluate its reproducibility.

## 2. Subjects and Methods

### 2.1. Study Population

A total of 500 women aged between 45 and 75 years, were randomly selected from the population register of 2001 of Ghent. A subgroup of 250 women was included in the validity study and asked to complete a FFQ and a 7 d EDR. In total, 142 FFQ and 78 EDR were collected, while 12 invitation letters were declared undeliverable. For the reproducibility study, a second sample of 250 women was chosen. They were asked to fill in the same FFQ twice (FFQ_1_ and FFQ_2_) with a 6 weeks interval [18]. Among them, 138 returned FFQ_1_ and 83 women completed FFQ_2_ as well; 17 invitation letters were declared undeliverable. A socio-demographic questionnaire was also administered.

### 2.2. Food-Frequency Questionnaire – Test Method

A self-administered semi-quantitative FFQ was developed to estimate the usual daily fat (total fat, SFA, MUFA, and PUFA), fibre, alcohol, caffeine, and theobromine intakes of Belgian post-menopausal women over the previous year. For the conceptualisation of this FFQ, the food consumption data of a survey in adult women [19] and knowledge from previously conducted population dietary surveys in Belgium were used. In total, 741 food items were aggregated into groups of conceptually similar foods based on their fat, fibre, alcohol, caffeine, and theobromine content per serving.

The final FFQ (Supplementary Material - Appendix 1) included 76 food groups contributing considerably to the total daily fat, fibre, alcohol, caffeine, and theobromine consumption. For each of these food groups the respondents were instructed to indicate the frequency and daily portion size categories that best fit their usual diet. The 6 frequency questions used (never or less than once a month; 1–3 d/month; 1 d/week; 2–4 d/week; 5–6 d/week; every day), were based on those advised by Willett [18]. Depending on the food group, 3–5 portion size categories were given, together with a list of common standard measures as examples. For some food groups, additional questions were asked regarding the type or preparation method, such as regular or decaffeinated coffee, and cooked or fried potatoes. When taking these additive questions into account, 157 food groups were listed in the FFQ.

All returned FFQ were reviewed for completeness by checking for multiple frequency answers, unmarked portion sizes, skipped food items and written comments. Although we suspect that some women skipped questions in the FFQ when they never consumed the particular product instead of indicating “never or less than once a month”, we preferred not to make any assumptions and did not replace these missing values. No FFQ of the validity study and 4 FFQ_2_ were excluded, since less than half of the questions had been answered. Data from the good-quality FFQ were processed using the scanning software package TELEForm (version 6.1, Cardiff Software Inc., San Marcos, California, USA). Nutritional values were assigned to each food group on the basis of weighted means of all aggregated items. The food composition data were based on the Belgian NUBEL [20] and the Dutch NEVO [21] food composition databases, the USDA national nutrient database [22], information from industries and literature [23–26]. The subject-specific total intakes of each nutrient studied, were computed by multiplying the specified frequency, portion size and nutritional value per 100 g product, and then summing for all food sources.

### 2.3. Estimated Diet Record – Reference Method

In the present study the reference method was a 7 d EDR, using diaries with a semi-structured, open entry format and consisting of 6 eating occasions (breakfast, morning snacks, lunch, afternoon snacks, dinner, and evening snacks). Detailed information on the type, including if possible brand names, and portion sizes, expressed as natural or household measures, of the food consumed during seven consecutive days [27] was collected. Separate sheets were enclosed for detailed descriptions of recipes, additional information, comments, or eating patterns not fitting in the diaries’ structure.

Dieticians carried out the exclusion procedure of the EDR. Only good-quality food diaries, including seven completed record days and containing sufficiently detailed descriptions of the food products and portion sizes consumed, were taken into consideration. As a cross-check, average energy, total fat, SFA, MUFA, PUFA, fibre, alcohol, caffeine, and theobromine consumption was calculated as the mean of the 7 recorded days. Diaries with very high or very low estimates were rechecked. In total, 14 EDR had to be excluded because of quality problems. The good-quality EDR were coded and entered in a Diet Entry and Storage program (BECEL Nutrient Calculation Program 5.03; Nederlandse Unilever Bedrijven B.V. Rotterdam, The Netherlands) using a standardised protocol from the Ghent University department of Public Health and a manual on food portions and household measures [28]. The same food composition databases as for the FFQ were consulted.

### 2.4. Socio-Demographic Questionnaire

In order to evaluate possible confounding factors and selection bias, a socio-demographic questionnaire, registering additive information about the women (country of birth, education level, smoking habits, *etc.*) was distributed. The participants were also asked to report their weight and height, which were used to calculate their BMI.

### 2.5. Data Collection

All correspondence was carried out by postal mail. As recommended by the EFCOSUM expert group [29], the invitation letters informed the participants about the aims of the study and asked to provide their written consent together with the completed (first) FFQ and socio-demographic questionnaire. The food diaries were distributed two weeks after collection of the FFQ. An interval of six weeks separated the first and the second FFQ administration in the reproducibility study. Detailed guidelines for completing both the FFQ and the food diaries, and examples were also provided. The fieldwork started in September 2005 and was finished in April 2006. Ethical approval was obtained by the Ethics Committee of Ghent University Hospital (EC UZG 2005/022).

### 2.6. Statistical analysis

Only good-quality EDR and FFQ were included in the analyses: the data of 64 women were useful for the validity analysis, while 79 participants delivered two complete FFQ for the reproducibility study. Power calculations [30] based on an α level of 0.01 and β of 0.05, showed that, with a sample size of 38 participants, we would be able to detect differences between the test and reference method for each nutrient similar to the intra-individual variations without generating statistically significant, but scientifically meaningless differences.

SPSS for Windows version 12.0 (SPSS Inc., Chicago, Illinois, USA) was used for all statistical analyses. Results were considered statistically significant at an α two-tailed level of 0.05. Tests for normality of the data were performed using the Kolmogorov-Smirnov test. Means and standard deviations (SD) of nutrient intakes, and differences between mean values obtained from the first and second dietary assessment were calculated. The paired Student’s *t*-test or the Wilcoxon’s matched-pairs signed-rank test was used to determine significant differences between means. Associations were described using Pearson’s correlation coefficients or non-parametric Spearman’s correlations. In the validity study, the correlation coefficients were deattenuated to correct for intra-individual variability, using the formula proposed by Beaton *et al.* [27]. The within-person variations were divided by the between-person variations to quantify the variance ratios λ_x_ of the 7 d food diaries.

Bland & Altman plots visualised the agreement between the test and reference method for each nutrient at an individual level [31]. In order to evaluate the questionnaire’s ability to assign individuals to the same categories of intake as the food diaries, all participants were classified into tertiles of nutrient intakes based on the distribution of data from the EDR and the FFQ [18]. Cross-classification analyses and weighted κ statistics calculated with a linear set of weights [32], were used to measure the level of agreement between the EDR and FFQ or between FFQ_1_ and FFQ_2_. The measurement error of the FFQ was analysed with the actual values for surrogate categories method as described by Willett [18]. The categories were compared using the one-way ANOVA or the Median test. The total fat, SFA, MUFA, PUFA, fibres, and alcohol estimates were compared to the recommended daily amounts for women proposed by the Belgian Health Council [33].

These recommendations were used as threshold values to define the specificity, sensitivity, and positive and negative predictive values of the FFQ, whereby intakes in line with the recommendations were defined as positive. In order to determine potential confounding factors for the validity and/or reproducibility of the FFQ, covariance analyses were performed with variables derived from the socio-demographic questionnaire. These variables were also used together with the dietary data to estimate the possible selection bias. Data of drop-outs and women excluded due to low-quality questionnaires or diaries were compared to those included in the statistical analyses with the independent Student’s *t*-test or via cross-tabulations with χ^2^ or Fisher’s exact tests.

## 3. Results

### 3.1. Validity Study

The mean age of the subjects included in the validity study (*n* 64) was 58 years. Thirty-seven women (54%) had a normal weight (18.5 kg/m^2^ ≥ BMI ≥ 24.9 kg/m^2^), whereas one woman (2%) had a BMI below 18.5 kg/m^2^ and 26 women (42%) were classified as overweight (BMI ≥ 25 kg/m^2^) and five of these (19%) were obese (BMI ≥ 30 kg/m^2^). A minority of the participants (*n* 11; 17%) were current smokers. Mean intakes of total fat, SFA, MUFA, PUFA, fibres, alcohol, caffeine, and theobromine estimated with the 7 d food diaries and FFQ, mean differences and deattenuated correlation coefficients between the test and reference method are presented in Table 1.

**Figure 1.**
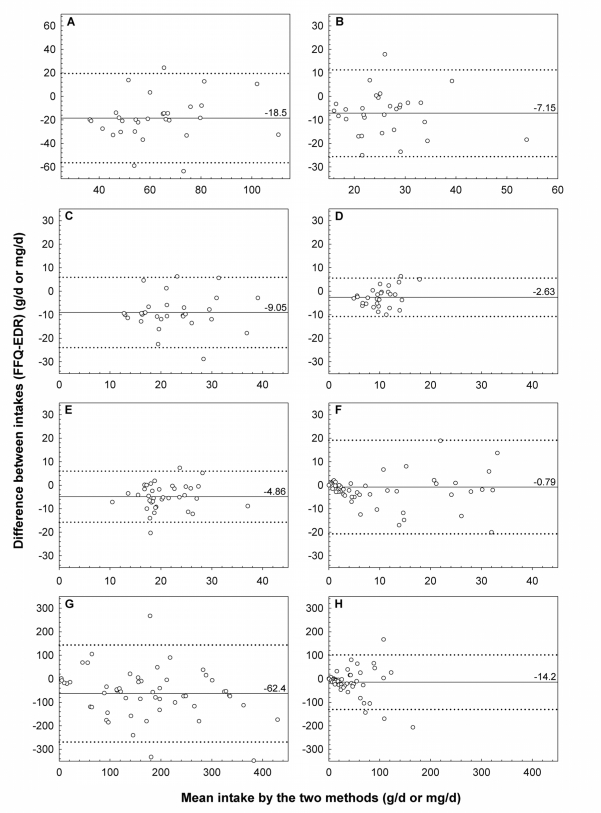
Bland & Altman plots visualising the differences between the mean intake of (A) total fat, (B) saturated fatty acids, (C) mono-unsaturated fatty acids, (D) poly-unsaturated fatty acids, (E) fibres, (F) alcohol, (G) caffeine, and (H) theobromine calculated from the 7d estimated diet record (EDR) and the food-frequency questionnaire (FFQ) (—— mean difference; ····· mean difference ± 2 SD).

Compared to the EDR, the FFQ underestimated the intakes of all nutrients analysed. Although the differences between the mean intakes were statistically significant, deattenuated correlation coefficients ranging from 0.47 (PUFA) to 0.94 (alcohol) were found between these methods. Deattenuation improved the correlation coefficients for all nutrients. The Bland & Altman plots of total fat, SFA, MUFA and PUFA estimates (Figure 1 A-D) were slightly divergent and showed a high degree of underestimation and acceptable limits of agreement. The differences in fibre consumption resulted in a similar pattern, but without divergence (Figure 1 E). Although good estimations were obtained for the intake of alcohol, caffeine, and theobromine, some outliners widened the limits of agreement and made the plots more divergent (Figure 1 F-H).

Cross-classification analyses showed that most subjects (43% for fibres – 73% for alcohol) were assigned to the same tertiles by both methods, whereas between 2% (alcohol) and 17% (SFA) were grossly misclassified (Table 2). The weighted κ values ranged from 0.25 for SFA to 0.66 for alcohol. Actual values for surrogate FFQ tertiles increased progressively in total fat, SFA, MUFA, alcohol, caffeine, and theobromine intakes over the surrogate categories (Table 3). Significantly different means were observed between the different tertiles for all nutrients except for total fat and SFA. Therefore, Fisher’s multiple comparison test was performed on the latter nutrients, revealing a significant difference (*P* 0.044) between the means of total fat intake of the extreme tertiles.

The mean differences between the FFQ and EDR were used as correction factors in the assessment of the specificity, sensitivity, and positive and negative predictive values of the FFQ for total fat, SFA, MUFA, PUFA, fibres, and alcohol estimates (Supplementary Material - Appendix 2). No significant confounding factors for the relative validity of the FFQ were found in the covariance analyses. The socio-demographic characteristics and dietary estimates were not significantly different between the women included in the analysis and the drop-outs and excluded participants.

### 3.2. Reproducibility Study

Participants of the reproducibility study (*n* 79) were on average 59 years old. Forty-five (57%) women had normal weight (18.5 kg/m^2^ ≥ BMI ≥ 24.9 kg/m^2^), whereas one woman (1%) had a BMI below 18.5 kg/m^2^ and 33 women (42%) were classified as overweight (BMI ≥ 25 kg/m^2^) and eight of them (24%) were obese (BMI ≥ 30 kg/m^2^). Only eight participants (10%) were current smokers.

The mean daily intakes of total fat, SFA, MUFA, PUFA, fibres, alcohol, caffeine, and theobromine obtained from the first and second FFQ were not significantly different and correlation coefficients ranging from 0.71 (caffeine) to 0.87 (alcohol) were obtained (Table 4). The percentages of subjects classified into the same and opposite tertiles are summarised in Table 5. Cross-classification showed no severe misclassification for MUFA and PUFA consumption. The weighted κ statistic ranged from 0.33 for fibres to 0.80 for MUFA. The covariance analyses revealed no significant confounding factors for the reproducibility of the FFQ. No significant differences in socio-demographic characteristics nor dietary estimates were found between the women included in the analysis and the drop-outs and excluded participants.

## 4. Discussion

A novel self-administered semi-quantitative FFQ was designed and validated in the context of intervention trials investigating dietary factors associated with the microbial metabolism of phyto-oestrogens in post-menopausal women [14, 15], and responding to the need for estimates of usual daily total fat, SFA, MUFA, PUFA, fibre, alcohol, caffeine, and theobromine intakes among Belgian women between 45 and 75 years old.

The validity of the FFQ was evaluated using different approaches. Comparison of means and Bland & Altman analyses revealed a tendency of the FFQ to underestimate the mean intakes measured by the EDR, especially for total fat, SFA, MUFA, PUFA, and fibres. Since our FFQ was not designed to estimate energy intake, we could not determine whether this was due to underreporting. Large standard deviations of the mean differences between the test and reference method were visualised in the Bland & Altman plots of alcohol, caffeine, and theobromine, suggesting that the use of the FFQ to estimate absolute intakes by individuals is not appropriate. In addition, a systematic increase in measurement error with increasing absolute intake of these components was observed. However, the primary goal of this instrument was to classify and rank subjects according to their nutrient intakes rather than achieving accurate results in terms of individual consumption.

Dietary instruments should have correlation coefficients of at least 0.40 and optimally in the range of 0.50–0.70 in order to reliably rank persons [18]. Thus, the observed deattenuated correlations (0.47–0.94) indicate that our FFQ has a realistic and desirable level of precision and a good ranking ability. The high proportions of participants cross-classified in the same or adjacent tertiles, between 83% for SFA and 92% for alcohol, confirm this. Based on the weighted κ values [32], the levels of agreement between the FFQ and EDR were fair for total fat, SFA, MUFA, PUFA, fibre, and theobromine, moderate for caffeine, and good for alcohol estimates. Given the results of the actual value for surrogate categories analyses, we could conclude that the FFQ can reliably distinguish extreme intakes for all nutrients under study, except for SFA.

The FFQ should not be used at an individual level (like in dietary counselling) for estimating the consumption of PUFA with the Belgian Health Council guidelines for women [33] as reference values, because 30% of the women would miss a required intervention, while 23% would be provided with an unneeded intervention. The specificity and sensitivity errors of the FFQ for the other nutrients under investigation were in the more acceptable range of 7–13% and 5–13%, respectively. Nevertheless we do not intend to use this FFQ to get correct absolute levels of intake, but to compare intakes of groups of subjects in a research setting.

Good reproducibility was established for the FFQ. No significant differences were found between the first and second administration. The high correlation coefficients (0.71–0.87) indicate that the random response error, sometimes due to lack of interest or motivation of the respondents or lack of clarity of the questionnaire, was rather small. The agreement [32] between the repeated administrations was fair for fibres, moderate for SFA, PUFA, and caffeine, and good for total fat, MUFA, alcohol, and theobromine, with a maximal misclassification of 7% for fibres.

The results of the present validation study compare favourably with those of other researchers who validated FFQ-derived fat (total fat, SFA, MUFA, and PUFA), fibre, and alcohol estimates relative to 24 h dietary recalls [17,34,35], a 3 d EDR [36] or two 7 d diaries [37]. Compared to our results, both Olafsdottir *et al.* [35] and Männistö *et al.* [37] reported for all these nutrients lower correlation coefficients of validity and reproducibility. The participants of the latter studies were young Icelandic (36±5 years) and Finnish women (51±9 years), respectively. Paalanen *et al.* [36] suggested that older Finnish women (50–79 years) score better than younger women because their dietary habits are more regular and therefore easier to report. The results of this subgroup were very similar to ours and much better than those among older men (50–79 years). On the other hand, slightly better correlations were achieved in the study of Kroke *et al.* [34] involving German men and women between 35 and 76 years old. In all studies, the poorest results were obtained for the intake of PUFA. This may be partly due to subjects’ desire to achieve social acceptance by emphasizing the use of foods considered to be healthy, such as fish. Another possible explanation could be the high within-subject variance for PUFA intakes, caused by the fact that PUFA are often concentrated in foods such as seafood, which are not always consumed on a daily basis [38]. Finally, PUFA are present in low concentrations in individual foods, but accumulate to significant levels in the context of a whole diet [38], therefore it is more difficult to inquire about this nutrient with a delimited questionnaire. Conversely, alcohol was the best scoring nutrient in all validation studies, probably due to the high consumer awareness related to his consumption of alcoholic beverages. Furthermore, the alcohol percentage of food and beverages is well-known and it is easy to aggregate and list the different major sources.

Unlike their results for fat, fibre, and alcohol estimates, Jain *et al.* [17] found higher correlation coefficients for the validity and reproducibility of caffeine intake in their study with Canadian women (54±14 years) than ours. In contrast, our correlations were better than those reported on coffee and tea consumption in Italian women (median age 52 year) [39]. Despite coffee being the major source of caffeine intake, a significant underestimation occurs when coffee is used as a surrogate measure for caffeine intake [40]. In the present study, the consumption of coffee, decaffeinated coffee, tea, and food and beverages containing chocolate were measured to approximate the intake of caffeine and theobromine. To our knowledge, this is the first validation study considering FFQ-derived theobromine estimates. It is important to note that the reliability of caffeine and theobromine estimates is questionable because many factors such as agricultural practices, geographical origin, post-harvest processing, and brewing methods [25], affect the amount of these components in foods and beverages. There is also a large fluctuation in how a same person prepares coffee, tea, and chocolate milk.

In intervention studies, dietary assessments often rely on a self-administered FFQ aiming at the assessment of usual long-term consumption and designed to rank subjects into quantiles of dietary intake. The issue of how to evaluate the accuracy and precision of a new dietary instrument is frequently debated. The problem with validation approaches in which a second dietary assessment method is used as reference, is that both methods may be biased and contain correlated errors. Biochemical markers reflecting dietary intakes are valuable tools since they do not rely on self-reports and their random measurement errors are not likely to correlate with those of dietary assessment methods. Unfortunately, no useful biomarkers were available for this validation study. Therefore, estimated diet records were chosen as reference method. Although both methods are subjected to a degree of misreporting, the measurement errors of the EDR and FFQ are highly independent, since, unlike the FFQ, the EDR method does not depend on memory, is open-ended, and involves direct estimation of portion sizes [41].

Weighted diet records are more accurate in terms of individual intakes, but estimated records achieve the same order of accuracy when ranking subjects and have a lower respondent burden [42]. Therefore, the estimated technique was preferred. Structured diaries guide the participants to report all their consumptions, even the easily forgotten snacks. Unfortunately, using an universal structure fitting everybody’s eating pattern is unfeasible, and therefore, some subjects might be influenced or unable to report their dietary habits correctly within this structure. In this study, semi-structured EDR were chosen, giving participants with an eating pattern not fitting within the diaries’ structure, the opportunity to use the blank sheets enclosed. In order to deal with day-to-day variation and to cover all days of the week equally, food diaries with seven consecutive recording days were used. However, problems such as declining accuracy of recording due to increasing fatigue and boredom, and potential alterations of dietary habits are intrinsic to long recording periods and contrast with the theoretical improvement of the precision of a measurement with increasing numbers of replicates (recording days) [43]. Although the FFQ referred to the year preceding the administration, seasonal variation in food consumption could not be considered since the validation study was carried out from September to April, however, the dietary intervention trials with phyto-oestrogens [14, 15] were also conducted during autumn and wintertime. Because the performance of a dietary assessment instrument depends on the characteristics of the study population and considering the target population in which the FFQ will be used, women between 45 and 75 years old living in the region of Ghent, were recruited. Sampling of subjects leads unavoidably to some selection bias: volunteers are not representative of the general population, as they are generally more concerned with health and diet than others, but forcing non-motivated individuals to participate in a study might influence the quality of the data as well [29]. As all correspondence was carried out by postal mail, it is unknown how many invitation letters reached their addressee and therefore no exact participation rates could be determined, yet we recognise these were probably rather low. Although the EFCOSUM expert group supports the choice of a population register as sampling frame [29], we could not access a recently updated list (2001 vs. 2005), and, considering the age of the target population, it is not unlikely that this non-coverage problem resulted in a high proportion of ineligibles due to migration or decease. There were no cases of non-response due to explicit refusal by a subject upon invitation. Our stringent inclusion criteria reduced the total number of women included in the validity and reproducibility analyses. However, no significant differences in socio-demographic characteristics nor dietary estimates were found between the women included, and the drop-outs and excluded participants. In summary, the results of the present validation study demonstrate the suitability of the FFQ to rank subjects according to their usual daily intakes of total fat, SFA, MUFA, PUFA, fibres, alcohol, caffeine, and theobromine. Additionally, the reproducibility of this FFQ was good.

## Figures and Tables

**Table 1.** Usual daily intakes of total fat, saturated, mono-, and poly-unsaturated fatty acids, fibres, alcohol, caffeine, and theobromine calculated from the 7 d estimated diet records and the food-frequency questionnaire; differences and deattenuated correlation coefficients between the test and reference method (*n* 64).

	7d EDR		FFQ		*P*	FFQ-EDR		Correlation	
	Mean	SD	Mean	SD		Mean	SD	*r*	*P*
Total fat (g/d)	73.2	24.7	53.1	21.2	0.001μ	–18.5	19.0	0.60‡	0.002
Saturated FA (g/d)	30.1	12.1	22.6	8.64	0.001μ	–7.15	9.23	0.51‡	0.008
Mono-unsaturated FA (g/d)	27.8	10.9	17.8	7.89	0.001μ	–9.05	7.45	0.60‡	0.002
Poly-unsaturated FA (g/d)	11.6	3.97	8.81	4.20	0.001μ	–2.63	4.06	0.47‡	0.030
Fibres (g/d)	22.0	5.85	18.3	5.85	0.001μ	–4.86	5.42	0.57‡	0.001
Alcohol (g/d)	9.90	11.0	9.07	13.5	0.012†	–0.790	9.97	0.94#	0.001
Caffeine (mg/d)	206	121	143	105	0.022μ	–62.4	103	0.64‡	0.001
Theobromine (mg/d)	48.1	63.3	31.9	41.1	0.001†	–14.2	58.9	0.57#	0.001

EDR, estimated diet record; FFQ, food-frequency questionnaire; FA, fatty acids;

^μ^ Paired Student’s *t*-test;

^†^ Wilcoxon’s matched-pairs signed-rank test;

^‡^ Pearson’s correlation coefficient deattenuated for within-individual variation;

^#^ Spearman’s correlation coefficient deattenuated for within-individual variation

**Table 2.** Cross-classification and weighted κ values of the 7d estimated diet records and food-frequency questionnaire tertiles of usual daily total fat, saturated, mono-, and poly-unsaturated fatty acid, fibre, alcohol, caffeine, and theobromine intakes.

	Percentage classified in		Weighted κ (95% lower, upper CI)
	Same tertile	Opposite tertile	
Total fat	53	13	0.32 (0.07, 0.58)
Saturated fatty acids	50	17	0.25 (0.00, 0.50)
Mono-unsaturated fatty acids	57	10	0.40 (0.15, 0.65)
Poly-unsaturated fatty acids	57	10	0.40 (0.15, 0.65)
Fibres	43	8	0.26 (0.03, 0.49)
Alcohol	73	2	0.66 (0.48, 0.84)
Caffeine	64	6	0.53 (0.34, 0.72)
Theobromine	53	6	0.40 (0.21, 0.59)

**Table 3.** Actual values for surrogate tertiles of usual daily total fat, saturated, mono-, and poly-unsaturated fatty acid, fibre, alcohol, caffeine, and theobromine intakes with the food-frequency questionnaire and the 7 d estimated diet records as surrogate and reference method, respectively.

	1^st^ tertile		2^nd^tertile		3^rd^ tertile		*P*
	Mean	SD	Mean	SD	Mean	SD	
Total fat (g/d)	61.8	11.7	74.8	14.4	78.2	23.6	0.103μ
Saturated fatty acids (g/d)	26.3	7.30	30.1	6.15	32.8	12.8	0.303μ
Mono-unsaturated fatty acids (g/d)	22.0	4.16	27.4	7.18	31.7	8.54	0.017μ
Poly-unsaturated fatty acids (g/d)	9.33	2.45	12.7	3.00	12.3	2.34	0.015μ
Fibres (g/d)	21.6	4.09	20.6	3.96	27.3	5.53	0.002μ
Alcohol (g/d)	1.43	3.37	5.03	5.08	21.8	9.52	0.001†
Caffeine (mg/d)	130	105	168	71.4	314	122	0.001μ
Theobromine (mg/d)	22.1	35.1	53.1	51.6	61.8	60.0	0.016†

^μ^ One-way ANOVA test;

^†^ Median test

**Table 4.** Usual daily intakes of total fat, saturated, mono-, and poly-unsaturated fatty acids, fibres, alcohol, caffeine, and theobromine estimated after the first and the second administration of the food-frequency questionnaire; differences and correlation coefficients between the food-frequency questionnaire (*n* 79).

	FFQ_1_		FFQ_2_		*P*	FFQ_1_-FFQ_2_		Correlation	
	Mean	SD	Mean	SD		Mean	SD	*r*	*P*
Total fat (g/d)	56.8	17.8	54.1	26.8	0.544μ	2.40	18.0	0.76‡	0.001
Saturated FA (g/d)	23.8	8.32	22.3	11.2	0.681μ	0.728	8.20	0.72‡	0.001
Mono-unsaturated FA (g/d)	18.3	6.53	18.2	8.75	0.520μ	0.789	5.79	0.78‡	0.001
Poly-unsaturated FA (g/d)	9.19	3.48	9.06	4.50	0.277μ	0.679	2.85	0.78‡	0.001
Fibres (g/d)	22.9	7.52	19.3	7.37	0.159μ	1.48	5.31	0.79‡	0.001
Alcohol (g/d)	7.22	8.55	8.29	9.85	0.942†	–0.709	5.36	0.87#	0.001
Caffeine (mg/d)	120	95.6	115	106	0.972μ	–0.367	77.1	0.71‡	0.001
Theobromine (mg/d)	50.2	63.5	46.8	63.2	0.760†	–3.00	35.0	0.79#	0.001

FFQ, food-frequency questionnaire;

FA, fatty acids;

^μ^ Paired Student’s *t*-test;

^†^ Wilcoxon’s matched-pairs signed-rank test;

^‡^ Pearson’s correlation coefficient;

^#^ Spearman’s correlation coefficient

**Table 5.** Cross-classification and weighted κ values of the first and second food-frequency questionnaire tertiles of usual daily total fat, saturated, mono-, and poly-unsaturated fatty acid, fibre, alcohol, caffeine, and theobromine intakes.

	Percentage classified in		Weighted κ (95% lower, upper CI)
	Same tertile	Opposite tertile	
Total fat	75	5	0.66 (0.35, 0.97)
Saturated fatty acids	68	5	0.58 (0.28, 0.88)
Mono-unsaturated fatty acids	83	0	0.80 (0.51, 1.09)
Poly-unsaturated fatty acids	64	0	0.58 (0.28, 0.88)
Fibres	48	7	0.33 (0.07, 0.60)
Alcohol	83	3	0.78 (0.61, 0.95)
Caffeine	64	2	0.57 (0.38, 0.76)
Theobromine	68	2	0.62 (0.43, 0.81)

## Supplementary Material - Appendix 1

### Food-frequency questionnaire

In this food-frequency questionnaire we inquire about your food habits of the previous year.

In the following table a variety of food products (food groups) is listed. Please describe (as exact as possible) how often you eat or drink the listed products and indicate the average daily portion. Consider also the meals taken away from home.

### How often (frequency)?

In the column with the heading ‘How often do you consume the following products?’ there are 6 possible answers.

Indicate your choice by colouring the circle near the answer that is most suitable for you.

### How much?

In the column with the heading ‘and what is the average portion per day?’ 3, 4 or 5 portion size options are given.

In the column with the heading ‘Example portion size’, a number of directive weights and measures are given. These can help you to quantify the average portion sizes.

Indicate your choice by colouring in the circle near the answer that is most suitable for you.

### Which type do you usually use?

In the last column you should indicate for some food products (food groups) the type or preparation method you usually use. Please choose only one answer, unless options are equally frequent. In the latter case you may indicate two options.

In case you would fill in the wrong option, you may cross it out and colour another option. Please indicate in such case the right answer with an arrow. Make sure you always fill in something, even when you consume a product rarely or never. In such case, choose the option ‘never or less than once a month’ without indicating a portion size or type.

### Example

Consider the following example: a person drinks every morning a cup of coffee (with caffeine) at home and a cup of herbal tea at work (5 days a week). During the weekend he/she takes a cup of English tea instead of herbal tea.

**Table:** Example Food-frequency questionnaire

Food groups	How often do you consume the following product?	and what is the average portion per day?	Example portion sizes	Which type do you usually use?
Coffee	○ never or less than once a month ○ 1–3 days per month ○ 1 day per week ○ 2–4 days per week ○ 5–6 days per week • every day	• 200 mL or less ○ between 200 – 400 mL ○ between 400 – 600 mL ○ 600 mL or more	*1 cup: 125 mL* *1 beaker: 225 mL*	• with caffeine ○ with reduced caffeine ○ without caffeine
Tea	○ never or less than once a month ○ 1–3 days per month ○ 1 day per week ○ 2–4 days per week ○ 5–6 days per week • every day	• 200 mL or less ○ between 200 – 400 mL ○ between 400 – 600 mL ○ 600 mL or more	*1 cup: 125 mL* *1 beaker: 225 mL*	○ regular English tea ○ green tea • herbal tea

**Table:** Food-frequency questionnaire

Food groups	How often do you consume the following product?	and what is the average portion per day?	Example portion sizes	Which type do you usually use?
Coffee	○ never or less than once a month ○ 1–3 days per month ○ 1 day per week ○ 2–4 days per week ○ 5–6 days per week ○ every day	○ 125 mL or less ○ between 125 – 250 mL ○ between 250 –375 mL ○ between 375 – 500 mL ○ 500 mL or more	*1 cup: 125 mL* *1 beaker: 225 mL*	○ with caffeine ○ with reduced caffeine ○ without caffeine
Tea	○ never or less than once a month ○ 1–3 days per month ○ 1 day per week ○ 2–4 days per week ○ 5–6 days per week ○ every day	○ 125 mL or less ○ between 125 – 250 mL ○ between 250 – 375 mL ○ between 375 – 500 mL ○ 500 mL or more	*1 cup: 125 mL* *1 beaker: 225 mL*	○ regular English tea ○ green tea ○ herbal tea
Milk/coffee cream in coffee/tea	○ never or less than once a month ○ 1–3 days per month ○ 1 day per week ○ 2–4 days per week ○ 5–6 days per week ○ every day	○ 8 mL or less ○ between 8 –16 mL ○ between 16 – 24 mL ○ 24 mL or more	*1 portion pot of coffee cream: 8 mL* *1 coffee spoon: 5 mL* *1 tablespoon: 10 mL*	○ coffee cream ○ skimmed milk ○ semi-skimmed milk ○ whole milk
Beer	○ never or less than once a month ○ 1–3 days per month ○ 1 day per week ○ 2–4 days per week ○ 5–6 days per week ○ every day	○ 200 mL or less ○ between 200 – 400 mL ○ between 400 – 600 mL ○ between 600 – 800 mL ○ 800 mL or more	*1 bottle/glass: 250 or 330 mL* *1 can: 330 or 500 mL*	○ non-alcoholic ○ strong beer *(Duvel, trappist,…)* ○ other beer *(pilsner, Palm, Kriek,…)*
Wine/sparkling wine	○ never or less than once a month ○ 1–3 days per month ○ 1 day per week ○ 2–4 days per week ○ 5–6 days per week ○ every day	○ 200 mL or less ○ between 200 – 400 mL ○ between 400 – 600 mL ○ 600 mL or more	*1 glass of champagne: 100 mL* *1 glass of wine: 125 mL*	
Aperitif *(port, Pineau, Pisang,…)*	○ never or less than once a month ○ 1–3 days per month ○ 1 day per week ○ 2–4 days per week ○ 5–6 days per week ○ every day	○ 75 mL or less ○ between 75 – 150 mL ○ between 150 – 225 mL ○ 225 mL or more	*1 glass = 75 mL*	
Liqueur and spirits	○ never or less than once a month ○ 1–3 days per month ○ 1 day per week ○ 2–4 days per week ○ 5–6 days per week ○ every day	○ 35 mL or less ○ between 35 – 70 mL ○ between 70 – 105 mL ○ 105 mL or more	*1 glass: 35 mL*	
Soup	○ never or less than once a month ○ 1–3 days per month ○ 1 day per week ○ 2–4 days per week ○ 5–6 days per week ○ every day	○ 200 mL or less ○ between 200 – 400 mL ○ between 400 – 600 mL ○ 600 mL or more	*1 bowl: 250 mL* *1 beaker: 225 mL*	○ clear soup without additives ○ soup with legumes ○ other soup
Fruit/vegetable juices	○ never or less than once a month ○ 1–3 days per month ○ 1 day per week ○ 2–4 days per week ○ 5–6 days per week ○ every day	○ 150 mL or less ○ between 150 – 300 mL ○ between 300 – 450 mL ○ 450 mL or more	*1 glass: 150 mL* *1 carton: 200 mL* *1 bottle (Looza): 200 mL*	○ fruit juice ○ vegetable beverages
Yakult, Actimel and the like	○ never or less than once a month ○ 1–3 days per month ○ 1 day per week ○ 2–4 days per week ○ 5–6 days per week ○ every day	○ 65 mL or less ○ between 65 – 110 mL ○ between 110 – 175 mL ○ 175 mL or more	*1 Yakult: 65 mL* *1 Benecol: 70 mL* *1 Actimel/Becel* *ProActiv:* *100 mL* *1 Optifit: 125 mL*	○ Probiotic beverages *(Actimel, Yakult,…)* ○ with plant stanol esters *(Benecol, Danacol,…)* ○ other: ______________
Soy/milk drinks/shakes *(Dan’Up, Fristi,…)*	○ never or less than once a month ○ 1–3 days per month ○ 1 day per week ○ 2–4 days per week ○ 5–6 days per week ○ every day	○ 125 mL or less ○ between 125 – 250 mL ○ between 250 – 375 mL ○ 375 mL or more	*1 glass: 150 mL* *1 beaker: 225 mL* *1 soy drink:* *250 mL* *1 bottle Dan’Up: 600 mL*	○ soy drinks ○ milk drinks and shakes
Chocolate milk	○ never or less than once a month ○ 1–3 days per month ○ 1 day per week ○ 2–4 days per week ○ 5–6 days per week ○ every day	○ 125 mL or less ○ between 125 – 250 mL ○ between 250 – 375 mL ○ 375 mL or more	*1 cup: 125 mL* *1 beaker: 225 mL* *1 bowl: 250 mL* *1 glass: 150 mL* *1 carton: 200 mL*	○ ready-to-drink ○ homemade with skimmed milk ○ homemade with low-fat milk ○ homemade with whole milk
Milk	○ never or less than once a month ○ 1–3 days per month ○ 1 day per week ○ 2–4 days per week ○ 5–6 days per week ○ every day	○ 125 mL or less ○ between 125 – 250 mL ○ between 250 – 375 mL ○ 375 mL or more	*1 cup: 125 mL* *1 beaker: 225 mL* *1 bowl: 250 mL* *1 glass :150 mL*	○ buttermilk ○ skimmed milk ○ low-fat milk ○ whole milk
Fresh cheese	○ never or less than once a month ○ 1–3 days per month ○ 1 day per week ○ 2–4 days per week ○ 5–6 days per week ○ every day	○ 75 g or less ○ between 75 – 150 g ○ between 150 – 225 g ○ 225 g or more	*1 Petit Gervais: 55 g* *1 Petit Gervais maxi: 100 g* *1 Danio: 200 g*	○ skimmed fresh cheese ○ low-fat fresh cheese ○ whole fresh cheese
Yoghurt with fibres *(Activia with fibres,…)*	○ never or less than once a month ○ 1–3 days per month ○ 1 day per week ○ 2–4 days per week ○ 5–6 days per week ○ every day	○ 125 g or less ○ between 125 – 250 g ○ between 250 – 375 g ○ 375 g or more	*1 pot: 125 g*	○ skimmed yoghurt with fibres ○ low-fat yoghurt with fibres ○ whole yoghurt with fibres
Yoghurt with fruit	○ never or less than once a month ○ 1–3 days per month ○ 1 day per week ○ 2–4 days per week ○ 5–6 days per week ○ every day	○ 125 g or less ○ between 125 – 250 g ○ between 250 – 375 g ○ 375 g or more	*1 pot: 125 g* *1 cup: 125 g* *1 dish: 150 g* *1 bowl: 250 g*	○ skimmed fruit yoghurt ○ low-fat fruit yoghurt ○ whole fruit yoghurt
NOT enriched with fibres				
Normal/aromatis ed yoghurt	○ never or less than once a month ○ 1–3 days per month ○ 1 day per week ○ 2–4 days per week ○ 5–6 days per week ○ every day	○ 125 g or less ○ between 125 – 250 g ○ between 250 – 375 g ○ 375 g or more	*1 pot: 125 g* *1 cup: 125 g* *1 dish: 150 g* *1 bowl: 250 g*	○ skimmed yoghurt ○ low-fat yoghurt ○ whole yoghurt
WIHTOUT fruit, WITHOUT fibres				
Milk/soy-based desserts	○ never or less than once a month ○ 1–3 days per month ○ 1 day per week ○ 2–4 days per week ○ 5–6 days per week ○ every day	○ 100 g or less ○ between 100 – 200 g ○ between 200 – 300 g ○ 300 g or more	*1 pot pudding or soy dessert: 100 or 200 g* *1 dish: 150 g* *1 bowl: 250 g* *1 pot rice pudding 100 or 200 g*	○ ready-to-eat ○ homemade with skimmed milk ○ homemade with low-fat milk ○ homemade with whole milk
Chocolate mousse, ice cream, bavarois, tiramisu	○ never or less than once a month ○ 1–3 days per month ○ 1 day per week ○ 2–4 days per week ○ 5–6 days per week ○ every day	○ 50 g or less ○ between 50 – 100 g ○ between 100 – 150 g ○ 150 g or more	*1 pot chocolate mousse: 70 g* *1 scoop ice cream: 35 g* *1 serving tiramisu bavarois: 80 g*	○ bavarois ○ chocolate mousse ○ ice cream ○ tiramisu ○ other: _____________
Nut/seeds and nut/seed-paste *(peanut butter, sesame paste)*	○ never or less than once a month ○ 1–3 days per month ○ 1 day per week ○ 2–4 days per week ○ 5–6 days per week ○ every day	○ 15 g or less ○ between 15 – 30 g ○ between 30 – 45 g ○ 45 g or more	*10 pealed peanuts: 20 g* *1 tablespoon nuts: 25 g* *1 tablespoon peanut butter: 15 g*	
Olives	○ never or less than once a month ○ 1–3 days per month ○ 1 day per week ○ 2–4 days per week ○ 5–6 days per week ○ every day	○ 15 g or less ○ between 15 – 30 g ○ between 30 – 45 g ○ 45 g or more	*5 olives: 20 g*	
Dried fruit	○ never or less than once a month ○ 1–3 days per month ○ 1 day per week ○ 2–4 days per week ○ 5–6 days per week ○ every day	○ 15 g or less ○ between 15 – 30 g ○ between 30 – 45 g ○ 45 g or more	*1 prune, apricot, date:8 g* *1 tablespoon raisins: 12 g*	
Berries, blackberries, raspberries	○ never or less than once a month ○ 1–3 days per month ○ 1 day per week ○ 2–4 days per week ○ 5–6 days per week ○ every day	○ 40 g or less ○ between 40 – 80 g ○ between 80 – 120 g ○ 120 g or more	*1 dish: 100 g*	
Other fruit (fresh, canned, compote)	○ never or less than once a month ○ 1–3 days per month ○ 1 day per week ○ 2–4 days per week ○ 5–6 days per week ○ every day	○ 100 g or less ○ between 100 – 200 g ○ between 200 – 300 g ○ 300 g or more	*1 mandarin: 60 g* *1 kiwi: 75 g* *1 peach: 100 g* *1 apple, pear, banana, orange 130 g* *1 tablespoon compote 40 g*	
Chocolate and candy bars *(Balisto, Mars, Twix,…)*	○ never or less than once a month ○ 1–3 days per month ○ 1 day per week ○ 2–4 days per week ○ 5–6 days per week ○ every day	○ 25 g or less ○ between 25 – 50 g ○ between 50 – 75 g ○ 75 g or more	*1 Mignonette: 10 g* *1 chocolate bar or Mars, Snickers: 50 g* *1 ChaCha: 25 g*	○ chocolate with nuts ○ chocolate without nuts ○ candy bar with nuts *(Bounty, Snickers,…)* ○ candy bar without nuts
Confectionery with chocolate *(chocolate truffles, M&Ms, chocotoff)*	○ never or less than once a month ○ 1–3 days per month ○ 1 day per week ○ 2–4 days per week ○ 5–6 days per week ○ every day	○ 15 g or less ○ between 15 – 30 g ○ between 30 – 45 g ○ 45 g or more	*1 chocolate truffle: 15 g* *1 Bouchée: 25 g* *1 bag of M & M ’s: 45 g* *1 chocotoff: 9 g*	
NO candy bars				
Tart, fruit pie, apple turnover	○ never or less than once a month ○ 1–3 days per month ○ 1 day per week ○ 2–4 days per week ○ 5–6 days per week ○ every day	○ 80 g or less ○ between 80 – 160 g ○ between 160 – 240 g ○ 240 g or more	*1 apple turnover: 100 g* *1 cupcake: 80 g* *1 tart (cupcake or serving): 150 g*	○ apple turnover ○ tart with jam ○ pie, tart
NO biscuit with fruit				
Other pie	○ never or less than once a month ○ 1–3 days per month ○ 1 day per week ○ 2–4 days per week ○ 5–6 days per week ○ every day	○ 80 g or less ○ between 80 – 160 g ○ between 160 – 240 g ○ 240 g or more	*1 serving of pie: 120 g*	○ with whipped cream or cream butter ○ almond cake, mattentaart ○ flan, millefeuille, rice pie, cream puff
Cake *(Madeleine, Zebra,…)*	○ never or less than once a month ○ 1–3 days per month ○ 1 day per week ○ 2–4 days per week ○ 5–6 days per week ○ every day	○ 20 g or less ○ between 20 – 40 g ○ between 40 – 60 g ○ 60 g or more	*1 slice of cake or cupcake: 30 g*	
Pancakes	○ never or less than once a month ○ 1–3 days per month ○ 1 day per week ○ 2–4 days per week ○ 5–6 days per week ○ every day	○ 60 g or less ○ between 60 – 120 g ○ between 120 – 240 g ○ 240 g or more	*1 pancake: 60 g*	
Sweet snacks with fibres *(Grany, Evergreen, coconut biscuit)*	○ never or less than once a month ○ 1–3 days per month ○ 1 day per week ○ 2–4 days per week ○ 5–6 days per week ○ every day	○ 20 g or less ○ between 20 – 40 g ○ between 40 – 60 g ○ 60 g or more	*1 Grany: 30 g* *2 Evergreen cookies 40 g* *1 coconut biscuit: 15 g*	
Waffle/wafer	○ never or less than once a month ○ 1–3 days per month ○ 1 day per week ○ 2–4 days per week ○ 5–6 days per week ○ every day	○ 30 g or less ○ between 30 – 60 g ○ between 60 – 90 g ○ 90 g or more	*1 wafer: 10 g* *1 Kempische galet: 30 g* *1 waffle: 40 g* *1 Belgian waffle: 60 g*	○ with chocolate ○ without chocolate
Dry biscuits *(Petit Beurre, Boudoir, Café Noir, Maria, Pim’s,…)* NO snacks with fibres	○ never or less than once a month ○ 1–3 days per month ○ 1 day per week ○ 2–4 days per week ○ 5–6 days per week ○ every day	○ 15 g or less ○ between 15 – 30 g ○ between 30 – 45 g ○ 45 g or more	*3 Sultana biscuits: 45 g* *1 Choco As: 20 g* *1 Petit Beurre: 13 g* *1 slice of ginger-bread:23 g*	○ biscuits with fruit *(Sultana)* ○ with chocolate *(Choco As, Pim’s)* ○ without chocolate *(Boudoir, Petit Beurre)*
Other biscuits *(Prince, Sprits, spiced biscuits…)*	○ never or less than once a month ○ 1–3 days per month ○ 1 day per week ○ 2–4 days per week ○ 5–6 days per week ○ every day	○ 15 g or less ○ between 15 – 30 g ○ between 30 – 45 g ○ 45 g or more	*1 spiced biscuit: 7 g* *1 Sprits or Prince: 20 g*	○ with chocolate ○ without chocolate
Salty snacks *(crisps, salted biscuits, Ringlings,…)*	○ never or less than once a month ○ 1–3 days per month ○ 1 day per week ○ 2–4 days per week ○ 5–6 days per week ○ every day	○ 20 g or less ○ between 20 – 40 g ○ between 40 – 60 g ○ 60 g or more	*1 small packet of crisps:45 g* *1 Tuc biscuit: 3 g* *1 packet of Mama Mia’s: 100 g*	
Breakfast cereals: muesli and crispy muesli (cruesli) *(Kelogg’s Extra, Kwakies,…)*	○ never or less than once a month ○ 1–3 days per month ○ 1 day per week ○ 2–4 days per week ○ 5–6 days per week ○ every day	○ 30 g or less ○ between 30 – 60 g ○ between 60 – 90 g ○ 90 g or more	*1 bowl of muesli: 40 g*	○ muesli ○ cruesli
Breakfast cereals: All Bran and Weetabix	○ never or less than once a month ○ 1–3 days per month ○ 1 day per week ○ 2–4 days per week ○ 5–6 days per week ○ every day	○ 30 g or less ○ between 30 – 60 g ○ between 60 – 90 g ○ 90 g or more	*1 bowl All Bran: 40 g* *1 Weetabix: 20 g*	○ All Bran Plus ○ All Bran Flakes ○ All Bran Choco ○ Weetabix
Breakfast cereals: high-fibre flakes	○ never or less than once a month ○ 1–3 days per month ○ 1 day per week ○ 2–4 days per week ○ 5–6 days per week ○ every day	○ 30 g or less ○ between 30 – 60 g ○ between 60 – 90 g ○ 90 g or more	*1 bowl of flakes: 30 g*	○ Clusters ○ Fitness ○ Fruit ‘n Fibre
Beakfast cereals: other *(Cornflakes, Chocapic, Special K,…)*	○ never or less than once a month ○ 1–3 days per month ○ 1 day per week ○ 2–4 days per week ○ 5–6 days per week ○ every day	○ 30 g or less ○ between 30 – 60 g ○ between 60 – 90 g ○ 90 g or more	*1 bowl of cereals: 30 g*	○ with chocolate ○ without chocolate
Biscuit rusk, cracotte, knäckebröd, Swedish bread, rice wafer	○ never or less than once a month ○ 1–3 days per month ○ 1 day per week ○ 2–4 days per week ○ 5–6 days per week ○ every day	○ 10 g or less ○ between 10 – 20 g ○ between 20 – 30 g ○ 30 g or more	*1 biscuit rusk, rice wafer, Parovitta: 8 g* *1 cracotte: 6 g* *1 Swedish bread: 10 g*	○ brown or wholemeal bread ○ other (white bread, rice wafers)
Brioches	○ never or less than once a month ○ 1–3 days per month ○ 1 day per week ○ 2–4 days per week ○ 5–6 days per week ○ every day	○ 50 g or less ○ between 50 – 100 g ○ between 100 – 150 g ○ 150 g or more	*1 medium-sized brioche: 65 g*	
Sweet bread, soft bread rolls	○ never or less than once a month ○ 1–3 days per month ○ 1 day per week ○ 2–4 days per week ○ 5–6 days per week ○ every day	○ 30 g or less ○ between 30 – 60 g ○ between 60 – 90 g ○ 90 g or more	*1 soft bread roll: 40 g* *1 slice of a big bread: 30 g* *1 slice of a small bread: 20 g*	○ soft bread rolls ○ sweet bread
White bread/bread rolls/baguette	○ never or less than once a month ○ 1–3 days per month ○ 1 day per week ○ 2–4 days per week ○ 5–6 days per week ○ every day	○ 30 g or less ○ between 30 – 60 g ○ between 60 – 90 g ○ 90 g or more	*1 bread roll: 40 g* *10 cm baguette: 40 g* *½ baguette : 120 g* *1 slice of a big bread: 30 g* *1 slice of a small bread: 20 g*	
Brown and wholemeal bread/bread rolls/baguette	○ never or less than once a month ○ 1–3 days per month ○ 1 day per week ○ 2–4 days per week ○ 5–6 days per week ○ every day	○ 30 g or less ○ between 30 – 60 g ○ between 60 – 90 g ○ 90 g or more	*1 bread roll: 40 g* *10 cm baguette: 40 g* *½ baguette : 120 g* *1 slice of a big bread: 30 g* *1 slice of a small bread: 20 g*	○ brown bread/bread rolls/baguette ○ wholemeal bread
Spread fat	○ never or less than once a month ○ 1–3 days per month ○ 1 day per week ○ 2–4 days per week ○ 5–6 days per week ○ every day	○ 7 g or less ○ between 7 – 14 g ○ between 14 – 21 g ○ 21 g or more	*5 g per slice of bread or rusk* *8 g per bread roll*	○ extra low-fat (15–25%) *(Vitelma Minelma, Buttella light, Solight, Becel Essential, Alpro Line S,…)* ○ low-fat (35–45%) *(Vitelma Progress, Buttela Soja, Balade, Becel Control, Spring, …)*○ butter and margarine *(Fama, Planta, Roda,..)* ○ other:
Chocolate spread/sprinkles/flakes	○ never or less than once a month ○ 1–3 days per month ○ 1 day per week ○ 2–4 days per week ○ 5–6 days per week ○ every day	○ 15 g or less ○ between 15 – 30 g ○ between 30 – 45 g ○ 45 g or more	*15 g per slice of big bread* *10 g per slice of small bread*	○ chocolate sprinkles/flakes ○ chocolate spread
Jam	○ never or less than once a month ○ 1–3 days per month ○ 1 day per week ○ 2–4 days per week ○ 5–6 days per week ○ every day	○ 15 g or less ○ between 15 – 30 g ○ between 30 – 45 g ○ 45 g or more	*15 g per slice of big bread* *10 g per slice of small bread*	
Feta, goat’s cheese, Mozzarella	○ never or less than once a month ○ 1–3 days per month ○ 1 day per week ○ 2–4 days per week ○ 5–6 days per week ○ every day	○ 15 g or less ○ between 15 – 30 g ○ between 30 – 45 g ○ 45 g or more	*15 g per slice of big bread* *10 g per slice of small bread* *1 small block of Feta 5 g* *1 Mozzarella: 125 g*	
Cheese spread type Philadelphia	○ never or less than once a month ○ 1–3 days per month ○ 1 day per week ○ 2–4 days per week ○ 5–6 days per week ○ every day	○ 15 g or less ○ between 15 – 30 g ○ between 30 – 45 g ○ 45 g or more	*15 g per slice of big bread* *10 g per slice of small bread*	○ normal ○ with herbs/fruit/vegetables ○ low-fat
Melted cheese/cheese spread *(Ziz,…)*	○ never or less than once a month ○ 1–3 days per month ○ 1 day per week ○ 2–4 days per week ○ 5–6 days per week ○ every day	○ 15 g or less ○ between 15 – 30 g ○ between 30 – 45 g ○ 45 g or more	*15 g per slice of big bread* *10 g per slice of small bread*	○ double-cream ○ normal ○ low-fat
Hard and semi-hard cheese *(Gouda, Westlite, Passendale,...)*	○ never or less than once a month ○ 1–3 days per month ○ 1 day per week ○ 2–4 days per week ○ 5–6 days per week ○ every day	○ 20 g or less ○ between 20 – 40 g ○ between 40 – 60 g ○ 60 g or more	*1 slice of cheese (10x10 cm): 25 g*	○ regular ○ low-fat *(St-Maarten, Westlitle,…)*
Other cheese *(Brie, Boursin, Camembert, Roquefort,...)*	○ never or less than once a month ○ 1–3 days per month ○ 1 day per week ○ 2–4 days per week ○ 5–6 days per week ○ every day	○ 15 g or less ○ between 15 – 30 g ○ between 30 – 45 g ○ 45 g or more	*15 g per slice of big bread* *10 g per slice of small bread*	
Vegetarian bread spread	○ never or less than once a month ○ 1–3 days per month ○ 1 day per week ○ 2–4 days per week ○ 5–6 days per week ○ every day	○ 15 g or less ○ between 15 – 30 g ○ between 30 – 45 g ○ 45 g or more	*15 g per slice of big bread* *10 g per slice of small bread*	
NO nut paste				
Crab/chicken/… salad	○ never or less than once a month ○ 1–3 days per month ○ 1 day per week ○ 2–4 days per week ○ 5–6 days per week ○ every day	○ 20 g or less ○ between 20 – 40 g ○ between 40 – 60 g ○ 60 g or more	*15 g per slice of bread* *35 g per French roll* *75 g per ½ baguette*	
Fish products *(smoked fish, canned fish)*	○ never or less than once a month ○ 1–3 days per month ○ 1 day per week ○ 2–4 days per week ○ 5–6 days per week ○ every day	○ 30 g or less ○ between 30 – 60 g ○ between 60 – 90 g ○ 90 g or more	*1 slice of smoked salmon/halibut: 30 g* *1 young herring or roll mop: 80 g* *1 drained can of tuna: 100 g*	○ smoked fish ○ canned fish
Fatty meat products *(pâté, salami, ground bief,…)*	○ never or less than once a month ○ 1–3 days per month ○ 1 day per week ○ 2–4 days per week ○ 5–6 days per week ○ every day	○ 20 g or less ○ between 20 – 40 g ○ between 40 – 60 g ○ 60 g or more	*15 g per slice of bread*	
Low-fat meat products *(Filet d’Anvers, ham, chicken ham,…)*	○ never or less than once a month ○ 1–3 days per month ○ 1 day per week ○ 2–4 days per week ○ 5–6 days per week ○ every day	○ 20 g or less ○ between 20 – 40 g ○ between 40 – 60 g ○ 60 g or more	*15 g per slice of bread*	○ smoked meat products *(filet de Sax, smoked ham,…)* ○ other meat products
Eggs	○ never or less than once a month ○ 1–3 days per month ○ 1 day per week ○ 2–4 days per week ○ 5–6 days per week ○ every day	○ 1 piece or less ○ 2 pieces ○ 3 pieces or more		Preparation ○ with butter/margarine ○ without butter/margarine
Vegetarian products *(tofu, quorn, burgers,…)*	○ never or less than once a month ○ 1–3 days per month ○ 1 day per week ○ 2–4 days per week ○ 5–6 days per week ○ every day	○ 50 g or less ○ between 50 – 100 g ○ between 100 – 150 g ○ 150 g or more	*1 packet of tofu: 75 g* *1 small burger: 55 g* *1 big burger: 95 g*	○ coated with breadcrumbs ○ without breadcrumbs
				Preparation ○ with butter/margarine ○ without butter/margarine
NO legumes				
Shellfish/crustaceans/squids	○ never or less than once a month ○ 1–3 days per month ○ 1 day per week ○ 2–4 days per week ○ 5–6 days per week ○ every day	○ 50 g or less ○ between 50 – 100 g ○ between 100 – 150 g ○ 150 g or more	*1 tablespoon of shrimps: 20 g* *1 kg of mussels with shells: 200 g*	Preparation ○ with butter/margarine ○ without butter/margarine
Fish/fish sticks (fresh or frozen)	○ never or less than once a month ○ 1–3 days per month ○ 1 day per week ○ 2–4 days per week ○ 5–6 days per week ○ every day	○ 60 g or less ○ between 60 – 120 g ○ between 120 – 180 g ○ between 180 – 240 g ○ 240 g or more	*1 fish stick: 30 g* *1 serving of fish: 175 g*	○ fish with breadcrumbs or fatty fish *(herring, mackerel, eels, salmon)* ○ other fish Preparation ○ with butter/margarine ○ without butter/margarine
Poultry *(mince and sausage of poultry included)*	○ never or less than once a month ○ 1–3 days per month ○ 1 day per week ○ 2–4 days per week ○ 5–6 days per week ○ every day	○ 60 g or less ○ between 60 – 120 g ○ between 120 – 180 g ○ between 180 – 240 g ○ 240 g or more	*1 chicken nugget: 25 g* *1 chicken or turkey breast : 160 g* *1 chicken leg: 160 g (boneless)*	○ with breadcrumbs or skin ○ without breadcrumbs or skin
				Preparation ○ with butter/margarine ○ without butter/margarine
Fat meat: bacon/mince/burger/sausag es *(blood pudding included)* NO poultry	○ never or less than once a month ○ 1–3 days per month ○ 1 day per week ○ 2–4 days per week ○ 5–6 days per week ○ every day	○ 60 g or less ○ between 60 – 120 g ○ between 120 – 180 g ○ between 180 – 240 g ○ 240 g or more	*1 frying sausage: 130 g* *1 burger (ham-, cheese-): 130 g*	Preparation ○ with butter/margarine ○ without butter/margarine
Low-fat meat: chops/spare ribs/stew/lamb	○ never or less than once a month ○ 1–3 days per month ○ 1 day per week ○ 2–4 days per week ○ 5–6 days per week ○ every day	○ 60 g or less ○ between 60 – 120 g ○ between 120 – 180 g ○ between 180 – 240 g ○ 240 g or more	*1 pork or veal chop or 2 lamb chops: 150 g (boneless)* *1 serving of stew: 160 g*	Preparation ○ with butter/margarine ○ without butter/margarine
Lean meat: roast/steaks	○ never or less than once a month ○ 1–3 days per month ○ 1 day per week ○ 2–4 days per week ○ 5–6 days per week ○ every day	○ 60 g or less ○ between 60 – 120 g ○ between 120 – 180 g ○ between 180 – 240 g ○ 240 g or more	*1 steak: 175 g* *1 cordon bleu or schnitzel: 150 g*	Preparation ○ with butter/margarine ○ without butter/margarine
Pasta *(penne, spaghetti,...)*	○ never or less than once a month ○ 1–3 days per month ○ 1 day per week ○ 2–4 days per week ○ 5–6 days per week ○ every day	○ 40 g uncooked or less: = 100 g cooked or less ○ 40–80 g uncooked = 100–200 g cooked ○ 80 –120 g uncooked = 200–300 g cooked ○ 120–160 g uncooked = 300–400 g cooked ○ 160 g uncooked or more = 400 g cooked or more	*50 g uncooked pasta = 125 g cooked pasta* *1 tablespoon cooked pasta: 25 g*	○ wholemeal ○ other
Rice and other grains *(bulgur, quinoa,…)*	○ never or less than once a month ○ 1–3 days per month ○ 1 day per week ○ 2–4 days per week ○ 5–6 days per week ○ every day	○ 25 g uncooked or less = 62 g cooked or less ○ 25–50 g uncooked = 62–125 g cooked ○ 50– 75 g uncooked = 125–187 g cooked ○ 75–100 g uncooked = 187–250 g cooked ○ 100 g uncooked or more = 250 g cooked or more	*60 g uncooked rice = 150 g cooked rice* *1 tablespoon cooked rice: 25 g*	○ brown rice ○ white rice ○ other grains
Fried potatoes *(croquettes, French fries,..)*	○ never or less than once a month ○ 1–3 days per month ○ 1 day per week ○ 2–4 days per week ○ 5–6 days per week ○ every day	○ 100 g or less ○ between 100 – 200 g ○ between 200 – 300 g ○ 300 g or more	*20 French fries, 3*–*4 croquettes or 2 potatoes: 100 g* *1 medium-sized packet of French fries: 250 g*	
Potatoes (cooked, steamed, baked, mashed,...)	○ never or less than once a month ○ 1–3 days per month ○ 1 day per week ○ 2–4 days per week ○ 5–6 days per week ○ every day	○ 75 g or less ○ between 75 – 150 g ○ between 150 – 225 g ○ between 225 – 300 g ○ 300 g or more	*1 cooked potato: 50 g* *1 tablespoon of mashed potatoes 50 g*	Preparation ○ cooked/steamed ○ baked ○ mashed
Raw vegetables	○ never or less than once a month ○ 1–3 days per month ○ 1 day per week ○ 2–4 days per week ○ 5–6 days per week ○ every day	○ 60 g or less ○ between 60 – 120 g ○ between 120 – 180 g ○ between 180 – 240 g ○ 240 g or more	*1 serving of leafy vegetables:50 g* *1 tablespoon of shredded carrots 20 g* *1 tomato: 150 g*	
Corn/green/pea s/legumes *(haricots, beans, lentils,…)*	○ never or less than once a month ○ 1–3 days per month ○ 1 day per week ○ 2–4 days per week ○ 5–6 days per week ○ every day	○ 40 g or less ○ between 40 – 80 g ○ between 80 – 120 g ○ 120 g or more	*1 tablespoon of green peas or corn: 20 g* *1 tablespoon of cooked legumes: 30 g*	
Vegetables prepared without sauce	○ never or less than once a month ○ 1–3 days per month ○ 1 day per week ○ 2–4 days per week ○ 5–6 days per week ○ every day	○ 60 g or less ○ between 60 – 120 g ○ between 120 – 180 g ○ between 180 – 240 g ○ 240 g or more	*1 tablespoon of prepared vegetables: 30 g*	Preparation ○ with butter/margarine (stewed) ○ without butter/margarine (cooked, steamed)
NO legumes				
Vegetables prepared with sauce *(cauliflower in cheese sauce, creamed spinach)*	○ never or less than once a month ○ 1–3 days per month ○ 1 day per week ○ 2–4 days per week ○ 5–6 days per week ○ every day	○ 60 g or less ○ between 60 – 120 g ○ between 120 – 180 g ○ between 180 – 240 g ○ 240 g or more	*1 tablespoon of vegetables prepared in sauce: 30 g*	Preparation ○ sauce prepared with butter/margarine ○ sauce prepared without butter/margarine
Dressing *(mayonnaise, sauce cocktail, vinaigrette,…)* NO ketchup NO pickles	○ never or less than once a month ○ 1–3 days per month ○ 1 day per week ○ 2–4 days per week ○ 5–6 days per week ○ every day	○ 12 g or less ○ between 12 – 25 g ○ between 25 – 50 g ○ 50 g or more	*1 tablespoon of mayonnaise: 25 g* *1 coffee spoon of mayonnaise: 10 g* *1 tablespoon of vinaigrette 10 g*	○ low-fat dressing ○ regular mayonnaise ○ other sauce *(sauce cocktail, tartar)*
Warm sauces *(gravy, sauce mushrooms…)*	○ never or less than once a month ○ 1–3 days per month ○ 1 day per week ○ 2–4 days per week ○ 5–6 days per week ○ every day	○ 25 g or less ○ between 25 – 50 g ○ between 50 – 100 g ○ 100 g or more	*1 tablespoon of gravy: 12 g* *1 tablespoon of curry sauce: 20 g*	○ gravy, no fluid added ○ gravy, fluid added ○ sauce prepared with a roux
Cream in preparations *(in soup, sauce, on ice cream, in cappuccino...)*	○ never or less than once a month ○ 1–3 days per month ○ 1 day per week ○ 2–4 days per week ○ 5–6 days per week ○ every day	○ 15 g or less ○ between 15 – 30 g ○ between 30 – 45 g ○ between 45 – 60 g ○ 60 g or more	*1 tablespoon of whipping cream: 10 g* *1 blob of cream: 10 g* *1 portion of cream in a cappuccino 20 g* *1 portion of cream on ice cream: 30 g*	○ soy cream ○ low-fat cream ○ regular cream (30–40%fat)

**Date:** __/__/____

## Supplementary Material - Appendix 2.

Specificity (error), sensitivity (error), and positive and negative predictive values of the food-frequency questionnaire for the usual daily intake of total fat, saturated, mono-, and poly-unsaturated fatty acids, fibres, and alcohol. Intakes in line with the Belgian Health Council guidelines for women [33] were defined as positive. The underestimation by the FFQ was taken into account.

**Table:**

	Specificity	Sensitivity	Predictive value		Guidelines (g/d)
			Positive	Negative	
Total fat	80 (10)	80 (10)	80	80	< 67
Saturated fatty acids	33 (13)	83 (13)	83	33	< 22
Mono-unsaturated fatty acids	64 (13)	84 (10)	70	80	> 22
Poly-unsaturated fatty acids	68 (30)	36 (23)	65	40	12–22
Fibres	88 (11)	33 (5)	94	20	> 30
Alcohol	90 (7)	62 (8)	86	67	0
